# Oral health of people experiencing homelessness in London: a mixed methods study

**DOI:** 10.1186/s12889-023-16648-x

**Published:** 2023-09-04

**Authors:** Huda Yusuf, Ali Golkari, Sarah Kaddour

**Affiliations:** 1 Queen Mary University of London, Institute of Dentistry, Turner Street, London, E1 2AD UK; 2 Pathway Fellow, Pathway, 4th Floor East, 250 Euston Road, London, NW1 2PG UK

**Keywords:** Homelessness, Oral health, Quality of life

## Abstract

**Background:**

Those experiencing homelessness have significant health and oral health needs and are at the extreme of health inequalities. The aim of the study was to conduct an oral health needs assessment for those experiencing homeless in London and impacts on their oral health-related quality of life.

**Methods:**

The oral health needs assessment consisted of quantitative and qualitative methods. This included a survey questionnaire to assess perceived oral health needs, and oral health related quality of life. In addition, a focus group was conducted with 13 peer advocates.

**Results:**

Findings from the focus group revealed numerous challenges for homeless populations to maintain good oral health and access to dental care including mental ill-health, stigma, costs, and chaotic lifestyles. A response rate of 79% (*n* = 315) was achieved for the questionnaire survey. Results showed high levels of unmet dental needs and risky health behaviours including 60% reporting being smokers, 39% consuming high amounts of sugar and 52.4% brushing their teeth less than twice a day. More than a third (32.1%) had experienced toothache. The majority of respondents 80% (*n* = 224) were very or fairly concerned about their dental health. There were significant associations between perceived oral health status and oral health-related quality of life.

**Conclusion:**

Those experiencing homelessness in London were found to have high levels of unmet oral health needs, which significantly impacted on their oral health-related quality of life. Focusing on changing behaviours alone is insufficient and therefore addressing the structural determinants of homelessness is vital in improving oral and health outcomes of this vulnerable population.

**Supplementary Information:**

The online version contains supplementary material available at 10.1186/s12889-023-16648-x.

## Background

Homelessness is prevalent globally and those experiencing it have multi-morbidities and are at risk of premature mortality with extreme health inequalities. Tri-morbidity is a common feature among this population with many being at risk of poor physical and mental health compounded by drug and substance misuse [1].

The causes of homelessness are multi-factorial and complex with interactions between structural explanations including the wider determinants (e.g. poverty, housing, unemployment, social policies), the political environment and individualist explanations (poor physical and mental health, experience of violence, abuse and neglect, drug and alcohol problems, experience of care or prison) [2]. There is a strong and consistent association between trauma and homelessness [3].

Homeless populations are at the forefront of health inequalities with the majority experiencing poor oral health including increased risk of tooth decay, periodontal diseases, oral cancer, and poor access to dental services [4, 5]. These risks are further aggravated by social isolation, psycho-social factors, deprivation, and life-time trauma [6–8].

The COVID-19 pandemic has highlighted health inequalities and clinical vulnerabilities of those experiencing homelessness. Recognising the challenges in protecting this population during the pandemic, the UK government announced emergency funding ‘Everyone in’ scheme for £3.2 million to house adult rough sleeping in emergency accommodation in hotels in March 2020 [9]. Those experiencing homelessness were at risk of contracting the virus and spreading it to others with significant consequences due to underlying communicable and non-communicable diseases, living on the streets or in crowded spaces with challenges in social distancing and self-isolation.

There has been limited research in understanding the oral health needs of those experiencing homelessness especially in the context of a global pandemic. A large proportion of those experiencing homelessness (those sleeping rough on the streets) in London (almost 5000) were housed in hotels. A whole systems approach including health, public health, local authority, service providers, academics, the voluntary sectors worked in collaboration to protect this vulnerable population, which included conducting a health needs assessment and signposting to local health and social care services. This provided an opportunity for a comprehensive assessment of oral health needs whilst this population was being accommodated in designated hotels. This would highlight the oral health needs of those experiencing homelessness alongside co-designing and delivery of oral health promotion and dental services to address existing oral health inequalities in London. Therefore, the aim of this study was to:

1. assess the oral health needs of those experiencing homelessness in London.
2. explore the views and experiences of those who have experienced homelessness on their perceived oral health needs and access and utilisation of dental services.

## Methods

A mixed methods approach was adopted utilising both qualitative and quantitative methodologies to undertake the oral health needs assessment. An exploratory sequential design was adopted where qualitative data from a focus group with peer advocates were collected and analysed first, then quantitative data were collected [10]. The focus group explored the challenges with oral health which partially informed the design of the survey. All methods were carried out in accordance with relevant guidelines and regulations and this were approved by Public Health England ethics committee (NRO137). Information sheets and verbal information was provided by social care and dental professionals prior to data collection at least one week ahead of data collection. Written and informed consent were obtained from each participant. Participation in the study was voluntary, and confidentiality was maintained by conducting the survey in a private space. All data were anonymised.

### Focus group

A focus group was conducted with peer advocates from Groundswell, a voluntary organisation working with people with experience of homelessness. The majority of peer advocates had experienced homelessness and were now supporting those who are homeless to navigate health and social care services. A topic guide was developed which explored the perceived health and oral health needs, views, and experiences of peer advocates in accessing health care including barriers and facilitators, which lasted 60 min and was conducted face to face. This was based on a literature review and feedback from a researcher in the voluntary organisation.

#### Survey questionnaire

To assess the percieved oral health needs and impacts on oral health-related quality of life, a questionnaire survey was conducted with rough sleepers who were accommodated in hostels and hotels under the ‘Everyone In’ scheme, as part of a UK government scheme to protect rough sleepers from COVID-19 during the pandemic [9]. The estimated sample size was 400 based on 50% of the population having dental problems based on 5000 of the population housed in hotels, with 95% confidence interval and 5% error. Convenience sampling was conducted across London in areas of increasing or a higher prevalence of homelessness. The survey was conducted between July 2020 and Feburary 2021.

The questionaire survey consisted of four domains (demographics, health behaviours, quality of life and access to dental services), using validated questions from the Adult Dental Health Survey (ADHS) and the General Practice Patient Survey [11, 12]. See additional Supplementary file 1. The survey was shared with a researcher in the voluntary organisation to ensure that it was relevant. The survey was conducted by community dental services and members of the dental public health team. Staff training was provided to those conducting the survey to quality assure data collection and ensure adherence to survey protocol but also to ensure that those conducting the survey are sensitive to the needs of this vulnerable population. The survey responses were recorded by the interviewer on a secure encrypted laptop. Survey responses were captured on a secure digital survey platform, ‘Select Survey’ (ClassApps In Selectsurvey.net v5.0).

#### Oral Health Impact Profile (OHIP-14)

A shortened version of the Oral Health Impact Profile (OHIP-14) containing six domains and nine questions from the Adult Dental Health Survey was included to assess the impacts of oral health on individuals’ oral health-related quality of life [13]. These nine questions were chosen out of the 14 original questions to ensure its relevance to the homeless population (did not include questions on interrupted meals, difficulty in doing jobs, difficult to relax) and to reduce the burden of a long questionnaire on participants. Questions were analysed individually rather than summing up the scores, with the minimum and maximum possible score of each question being 1 and 5 respectively [13].

### Data analysis

#### Focus group with groundswell peer advocates

The focus group recording using a tape recording was transcribed and stored securely on the university’s cloud. Thematic analysis was used to analyse the data [14]. Thematic analysis, a method for identifying, analysing, and reporting patterns (themes) within data and involves constant moving backwards and forwards between the stages of analysis, and is therefore a cyclical rather than a linear process. One researcher analysed the data. Any discrepancies were discussed with the co-authors. Emerging themes within the data were identified and written down as a list of ideas by the margins. The second stage involved identifying initial themes/codes/sub-categories in the data that emerged or recurring themes. The third stage involved simultaneously examining the topic guide with the initial themes/codes that were identified earlier to identify the broader themes. A thematic chart was developed.

### Survey questionnaire

Results were analysed using SPSS version 28 (IBM Corp., NY, USA). Means and standard deviations are reported for each of the OHIP-14 related questions, grouping them within domains. To assess the normality of the distribution of OHIP-14 related questions, the Kolmogorov-Smirnov test was employed. The results indicated that none of the nine OHIP-14 related questions followed a normal distribution. Therefore, non-parametric Kruskal-Wallis tests were used to examine associations between concerns about oral health and different OHIP-14 questions. Additionally, Mann-Whitney U tests were conducted to explore associations between reported oral health problems and each OHIP-14 question. Sugar intake was combined for food and drinks and categorised into low sugar (five or less times per week) and high sugar intake (six times or more times per week). This was a similar method that was adopted by the Adult Dental Health Survey to allow comparisons to be made with the general population.

## Results

### Focus group with groundswell peer advocates

Thirteen peer-advocates who provided support to adults experiencing homelessness who had consented attended the focus group. In total, three major themes were identified; current health and oral health issues, trauma and isolation versus optimism.

#### Current health and oral health issues

The most common reported health condition was mental health followed by COPD, diabetes, respiratory diseases and oral health.

“Top of the list is mental health” (P3).

Oral health problems included toothache, abscesses, loose teeth and dentures with significant impacts on quality of life, especially feeling embarrassed and self-conscious about their appearance.

“After working with a guy for about one year… I knew a client for one year and I took him for many appointments and after he got his false teeth fitted, I saw him smile for the first time and I realised that I have never ever seen him smile… So, I can’t imagine what he was going through… looking back on it now every time he was having a giggle, he would be covering his mouth” (P10).

Maintaining oral health whilst living on the streets is challenging. Drug and alcohol use and smoking can put those experiencing homelessness at risk of poor oral and general health.

“They don’t maintain their oral health, they can’t … if you have to live with out-of-date xxxx and they are undernourished” (P3).

“We have a lot of our clients who are on methadone….in those 3 or 4 years their teeth have rotted because of the amount of sugar in methadone” (P5).

There is recognition that some of the those experiencing homelessness may have cognitive impairment or learning disabilities. Peer advocates recommended that clinicians should use simple language and being cognisant that not all individuals will be familiar with using digital technologies and aids for example, digital tablets.

#### Trauma and isolation

Some of the peer advocates reported that the psychological trauma and persistent isolation meant that they had a sense of wanting to be integrated back into society. Although they felt designated homeless health services were helpful, some preferred to access general health services like the rest of the general population. Some of the peer advocates reported that having specialist services may further aggravate their social isolation.

“Services need to understand that while our clients maybe stereotyped as aggressive, alcohol, drugs, serial DNA, the need for some kind of educational programme for people to understand the trauma behind the behaviour and develop some sympathy and empathy for the situation people find themselves in” (P7).

“These homeless specific practices I personally don’t agree with them, all practices should be doing it and also it reinforces the fact that they have an excluded lifestyle and that they are different…” (P2).

- Trust, relationships, and peer support

There was a strong consensus throughout the focus group on building trust and relationships. There was a lack of trust in health professionals due to negative stereotypes of homeless individuals, resulting in health professionals being judgmental and aggressive towards them. Having peer support was seen to be important in having their voices heard and supporting them to access relevant services as they had built trusting relationships with some dental practices.

“They have very low expectations of what they get in particular dentists. …They don’t know what they can get once they are in the journey, actually dealing with receptionists is very hard “(P2).

b. Bureaucracy

There are challenges in communication between health providers and those experiencing homelessness leading to a sense of isolation or being ignored. There is a lack of clarity on their entitlements, services unable to deal with addressing their chaotic lifestyles including not have a permanent address or access to mobile phones. There are significant challenges in maintaining contact and paying for costs of dental treatment.

“If you are homeless and you don’t have a phone and you are full of anxiety and trying to make a phone call when you are full of anxiety and you think nobody is going to listen to you” (P6).

c. Living in fear

A number of participants expressed fear of accessing health services due to their experience of degrading and disrespectful treatment by health professionals and administrative staff. Additionally, some were embarrassed about their mouths and expressed fear of the use of local anaesthetic for dental treatment.

“For me from my experience most people are scared of the dentist and not because of the needle but because they treat them bad. I am one of them.”

Some expressed fear of the needle due to prolonged drug abuse and felt that this further aggravated their general fears.

“There is a taboo around the green needle that great one and you get a dentist waving one of those around why do they have one of those big needles and it is associated with drugs. Why can’t you use a fine needle?”(P3).

#### Optimism

Some of the peer advocates reported the importance of oral health on self-esteem, confidence as a life changing experience. Despite having challenges in looking after their mouths when rough sleeping, they were keen to seek dental care to improve their oral health status in terms of function and aesthetics. It was perceived to be as an important step in reintegrating into society. The ideal service would be flexible, compassionate, and meet their health and social needs.

“People do want dental care and the transformative power of having your teeth fixed on somebody’s life is very misunderstood and…. it can change your whole, …it increases their smile and increases their confidence” (P11).

- Flexibility

Having recognised their chaotic and unpredictable lifestyles, they expressed the need for flexibility in accessing dental services. They also wanted less bureaucracy to encourage access to dental services. There seems to be a mismatch between organisation of dental services and appointments with the needs of those experiencing homelessness.

“It can be complex-I have heard it said for people who are drinking will need a drink in the morning and you have to get them before they have had too much to drink “ (P3).

b. Empathy, compassion, and kindness

The majority expressed the need for empathy and compassion. They wanted health professionals to listen to them, understand their needs and respect them as human beings, thus maintaining their dignity.

“The gentle dentist-when I was having painful treatment on one of my teeth, one of the nurses was holding my hand and I wasn’t expecting it” (P12).

In summary, although peer advocates reported difficulties in maintaining good oral health and challenges in accessing dental services, they highlighted the importance of good oral health and its transformational power on self-esteem and confidence.

### Questionnaire survey

#### Demographics of the sample

A total of 315 individuals (response rate 79%) completed the survey and were included in the analysis. The majority of responders were male (70.5%, *n* = 222), a third (31.4%, *n* = 99) of respondents were aged 35–44 years, with 1 in 5 (20.3%, *n* = 64) identifying themselves as White British (Table 1).

#### Reported health-related behaviours

Sugar consumption was prevalent with 55.6% (*n* = 175) of respondents reported that they consumed sugars (drinks and food) at least six times per week. In terms of toothbrushing, 45.1% (*n* = 142) brushed their teeth twice a day, 36.5% (*n* = 115) once a day and 15.9% (*n* = 50) less than once a day. In total, 60% (*n* = 189) of respondents reported that they were current smokers, with 23.2% (*n* = 73) stated to have never smoked. Sugar consumption was categorised into high or low sugar users, using answers derived from three variables. A small number of respondents (*n* = 50, 16.2%) reported being on methadone.

**Table 1:** Demographic characteristics of the sample and health-related behaviours

Characteristics of the sample	Number (%)
**Sex**	
Male	222 (70.5%)
Female	90 (28.6%)
Prefer not to say	3(0.9%)
**Age**	
Under 25	18 (5.7%)
25–34	67 (21.3%)
35–44	99 (31.4%)
45–54	69 (21.9%)
55–64	52(16.5%)
65 and over	10 (3.2%)
**Ethnicity**	
White British	64(20.3%)
White Irish	10(3.2%)
White other	58(18.4%)
White Gypsy or Irish Traveller	1(0.3%)
Black	85(27.0%)
Asian/Asian British	34(10.8%)
Mixed white and Black	8(2.5%)
Mixed white and Asian	7(2.2%)
Any other Ethnicity	43(13.7%)
Did not say	5 (1.6%)
**Health Behaviours**	
**Consumption of sugary foods**	
Low sugar intake	175 (55.6%)
High sugar intake	123 (39.0%)
Did not respond	17 (5.4%)
**Reported frequency of tooth brushing**	
Twice a day	142(45.1%)
Once a day	115 (36.5%)
Less than once a day	50 (15.9%)
Did not respond	8 (2.5%)
**Smoking status**	
Current smoker	189 (60.0%)
Past smoker	47 (14.9%)
Never smoked	73 (23.2%)
Did not respond	6 (1.9%)

+ Has cakes, biscuits, puddings or pastries, sweets or chocolate or fizzy drinks 6 or more times a week

#### Self-reported oral health problems

**Fig. 1:**
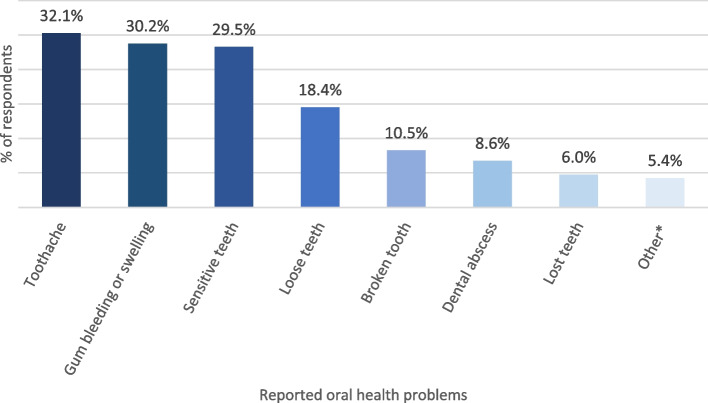
Self-reported dental problems stated by respondents (%). Other including ‘chipped teeth, lost fillings, pain, broken teeth and poor-fitting dentures’

When asked ‘how concerned are you about your dental health’, 78.1% (*n* = 246) of those who responded felt they were very concerned or fairly concerned about their dental health. Figure 1 shows the self-reported dental problems expressed by respondents. The most common reported oral health problem was toothache followed by bleeding or swollen gums, followed by sensitive or loose teeth.

We assessed the impacts of having an oral health problem on participants’ quality of life by analysing elements of OHIP-14. The most common impacts were being self-conscious (mean 3.0, SD 1.6) and being embarrassed (2.8, SD 1.6) followed by discomfort in eating (2.7, SD 1.5), aching pain (2.7, SD 1.4) and feeling tense (2.7, SD 1.5), respectively (Table 2).

**Table 2:** OHIP 14-items means and standard deviations

Domains	Items	N	Mean	SD
Functional Limitation	pronouncing words	300	1.9	1.3
	Taste	297	1.9	1.3
Physical Pain	Aching	296	2.7	1.4
	uncomfortable eating	289	2.7	1.5
Psychological Discomfort	self- conscious	294	3.0	1.6
	tense	290	2.7	1.5
Physical disability	unsatisfactory diet	294	2.4	1.5
Psychological disability	embarrassed	297	2.8	1.6
Handicap	unable to function	300	2.1	1.3

Perceived oral health was significantly associated with all six domains of quality of life (Table 3). This was a linear relationship where those who reported to be less concerned about their oral health were found to report less impact, compared to those who were fairly or very concerned, reporting significant impacts on handicap and psychological discomfort. Furthermore, having a dental abscess significantly affected all domains of quality of life. Toothache or pain’ bleeding or swollen gums were also associated with significant impacts on all aspects of quality of life(Table 4).

**Table 3:** Perceived oral health and impacts on quality of life (OHIP-14)

Impact	Level of concern	N	Mean	SD	*P*-value
**Pronouncing words**	Not concerned	60	1.4	0.8	< 0.001
	Fairly concerned	95	1.5	0.9	
	Very concerned	142	2.3	1.5	
	Total	297	1.9	1.3	
**Aching**	Not concerned	59	1.9	1.1	< 0.001
	Fairly concerned	94	2.5	1.3	
	Very concerned	141	3.2	1.4	
	Total	294	2.7	1.4	
**Uncomfortable eating**	Not concerned	59	1.9	1.2	< 0.001
	Fairly concerned	89	2.4	1.3	
	Very concerned	138	3.4	1.5	
	Total	286	2.9	1.5	
**Self-conscious**	Not concerned	57	1.8	1.2	< 0.001
	Fairly concerned	93	2.5	1.4	
	Very concerned	142	3.8	1.5	
	Total	292	3.0	1.6	
**Tense**	Not concerned	57	1.7	1.1	< 0.001
	Fairly concerned	92	2.2	1.2	
	Very concerned	139	3.5	1.5	
	Total	288	2.7	1.5	
**Unsatisfactory diet**	Not concerned	58	1.7	1.1	< 0.001
	Fairly concerned	94	1.8	1.1	
	Very concerned	139	3.1	1.	
	Total	291	2.4	1.5	
**Embarrassed**	Not concerned	60	1.8	1.3	< 0.001
	Fairly concerned	94	2.2	1.3	
	Very concerned	140	3.6	1.5	
	Total	294	2.8	1.6	
**Unable to function**	Not concerned	60	1.6	1.0	< 0.001
	Fairly concerned	95	1.61	0.914	
	Very concerned	142	2.69	1.498	
	Total	297	2.13	1.351	

**Table 4:** Association between oral health problems and various oral health-related quality of life domains using OHIP-14

Quality of life items (OHIP-14)	Type of current dental or oral problem ^a^						
	Toothache or pain	Gum bleeding or swelling	Sensitive teeth	Loose teeth	Broken tooth	Dental abscess	Lost teeth
Trouble pronouncing words	0.002	^b^	^b^	0.007	0.040	0.004	< 0.001
Taste change	0.026	< 0.001	^b^	< 0.001	^b^	< 0.001	^b^
Painful aching	< 0.001	< 0.001	0.009	< 0.001	^b^	< 0.001	^b^
Uncomfortable eating	< 0.001	< 0.001	0.016	0.002	^b^	< 0.001	0.034
Self-conscious	0.003	0.003	0.005	< 0.001	^b^	0.027	< 0.001
Felt tense	< 0.001	0.004	^b^	0.002	^b^	< 0.001	< 0.001
Unsatisfactory diet	< 0.001	0.013	0.048	0.004	0.006	< 0.001	< 0.001
Embarrassed	0.028	0.032	^b^	< 0.001	0.043	0.022	< 0.001
Unable to function	< 0.001	0.004	^b^	0.003	^b^	< 0.001	0.012

^a^The number of cases having other problems such as broken, loose, or lost dentures, lost filling, lost crown/bridge, and mouth ulcers were too few to run a valid statistical test

^b^Not statistically significant

#### Triangulation of the qualitative and quantitative data

Data triangulation was conducted by comparing the data from the focus group with the quantitative data from the oral health survey. This was conducted in order to ensure validation of the analysis, as well as obtaining a comprehensive picture of oral health needs of those experiencing homelessness. Data extracted from the focus group with peer advocates and from the questionnaire survey revealed that risky health behaviours were evident. Those who were sampled consumed sugary food and drinks regularly, with 52.4% brushing their teeth less than twice a day with a high prevalence of smoking. This was a consistent finding with the focus group where peer advocates reported challenges in maintaining oral health when living on the streets in terms of access to hygiene facilities and access to a healthy diet. It was perceived to be unrealistic to expect those who were sleeping rough on the streets to engage in healthy behaviours. There were divergent findings from the focus group and the survey questionnaire in relation to drugs consumption. In total, 16.2% of those who responded to the questionnaire reported taking methadone whereas peer advocates reported that drug use was common among those experiencing homelessness.

There was agreement that oral health was the second most prevalent condition after mental health. This was corroborated by the survey findings in which the majority of participants (78.1%) were either very concerned or concerned about their oral health. The most common reported problem both in the focus group and the survey questionnaire was toothache. Having poor oral health was associated with significant impacts on oral health-related quality of life especially in terms of psychological discomfort and disability and physical disability. This was in agreement with the findings from focus group in which peer advocates reported impacts on ability to eat, being self-conscious and feeling embarrassed. Some of the peer advocates strongly expressed the significance of good oral health on self-esteem and the ‘transformative power’ of teeth on physical and psych-social well-being. They had an optimistic approach with a desire to improve their oral health and to be integrated back into society.

## Discussion

This is one of the first studies using mixed methodology to assess the oral health of those experiencing homelessness. Triangulating the data from focus group and the health needs assessment conducted by the medical team at University College Hospital, oral health was second most prevalent health condition (30%) after mental health (50%).

The results from the questionnaire survey revealed that 41.3% of the sample were identified as White Other, 27% as Black and 10.8% Asian when compared with 53.8% being classified as White Other, 13.5% as Black, and 20.7% as Asian in the general population in London, respectively [15]. In total, 78.1% of participants reported that they were concerned or very concerned with their dental health. The most common reported dental problem was dental pain reported by 32.1% of respondents, followed by sensitive teeth (30.2%) and swollen or bleeding gums (29.5%). This is a similar finding to the general population and studies based in Scotland and in London among homeless populations [11, 16, 17]. Unhealthy behaviours were prevalent among the homeless population in London. Sugar consumption was high with 39% consuming sugary food and drinks on a regular basis. This is lower than expected when compared to other surveys [17]. However, this could have been impacted by the Everyone in scheme where those experiencing homelessness were provided with three meals a day in hotels during the pandemic and therefore reducing access and availability of sugary snacks. In relation to toothbrushing habits, 46.3% of respondents brushed their teeth twice a day and 36.5% brushing once a day, respectively. This is higher than that reported in the literature but much lower than the general population where 75% of adults reported brushing their teeth at least twice a day [11, 16]. This could be due to distribution of toothbrush and toothpaste packs during the Everyone in scheme. However, this could be partially explained by chaotic lifestyles and challenges in accessing oral hygiene products and facilities. Additionally, 60% were current smokers which is much higher than adults (13.3%) in the general population [18].

The majority of participants reported impacts of poor oral health on eating, speaking and feeling self-conscious and tense. This aligns with other studies [16, 17]. The findings have demonstrated considerable unmet oral health needs and physical pain with significant impacts on functional limitation, psychological discomfort, and disability and ultimately inability to function. Triangulation of the data from the focus group and the survey highlighted the importance of oral health for those experiencing homelessness and feeling of embarrassment being a significant impact on overall health and well-being, which should not be underestimated.

Studies have demonstrated that the prevalence of mental health disorders among those experiencing homelessness in high-income countries to be high affecting 76.2% (95% CI 64.0–86.6%) of the population. Mental health disorders included: depression, anxiety, and schizophrenia [19]. There is a well-established bi-directional association between mental health and oral health [20]. Individuals with a mental health condition may be at risk of poor oral health due to health-related behaviours and neglect, substance misuse and medication, resulting in poorer oral health outcomes especially among those with severe mental illness [20]. On the other hand, individuals suffering from severe mental illness may experience anxiety in receiving dental care resulting in dental phobia. Perception of dental pain may be heightened in those with depression [21].

Homelessness can result from trauma; a strong feature along the homelessness journey starting from pre-conception and into childhood [22]. This is further aggravated by being homeless itself is a traumatic experience with risk of losing family and friends, experiencing physical and sexual assault, and being social excluded and stigmatised. They may be fearful of accessing housing, employment, health, and social care resulting in a negative spiral of isolation and hopelessness. Therefore, trauma-informed care is recommended in engaging with those experiencing homelessness by recognising the impacts of trauma by providing an environment in which the individual is empowered and given control of their lives.

### Addressing the structural determinants of homelessness to tackle health and oral health inequalities

Improvements in oral health can not achieved by solely relying on modifying health behaviours or provision of health services. Prevention needs to be at the heart of homeless policies nationally and globally. Considering the complex physical, psychological and social needs, structural determinants for homelessness need to be addressed including access to good quality housing and multi-disciplinary working across health, education, employment and social sectors. A continuum of interventions is required upstream (legislation and public policy), mid-stream (creating supportive environments) and downstream, provision of health services that are tailored to the needs of this heterogenous population. Access to affordable housing with high quality wrap around care is therefore essential in moving individuals out of homelessness. Considering mental health as the most prevalent health issue, integrated care in which mental health and housing are jointly provided to homeless populations has been shown to be have a greater impact than providing mental health services alone [23, 24]. Several high income countries have developed public policies around homelessness including Finland, Australia, the US and Canada [24–28]. These mainly focus on provision of housing, high quality support with a focus on prevention of homelessness. Linking housing with mental health services is associated with reduction in psychiatric symptoms and less likelihood of returning to rough sleeping. One example of such initiatives is Housing First. This is an integrated model of care, which includes housing, health care, public health and social care, the voluntary sector working collaboratively to support those who are rough sleeping. Evidence from evaluation of the scheme in nine selected areas in England has shown that improvements in physical and mental health among users of the initiative [29].

The significance of oral health can not be under-estimated in terms of impacts on confidence and self-esteem. Results from the focus group and the survey questionnaire highlighted feeling of embarrassement and feeling conscious about the appearance of their teeth. This rehabilitation from being embarrassed to having a functioning and aesthetic dentition can support individuals to feel accepted by the rest of society and feel less stigmatised with improvements in psychosocial, functional, and physical well-being. There was agreement among peer advocates of the transformational power of teeth on a homeless individual’s confidence and self-esteem in being integrated back into society and living a “normal” life.

Therefore, dental services need to be holistic, flexible and available at different stages of homelessness. In terms of dental care, some individuals may only want to attend when experiencing dental problems and therefore flexible models of care need to be developed accordingly alongside routine dental services. Access to dental services needs to be readily available and accessible, accomodating with outreach and in reach models of care that are trauma-informed. The stigma and discrimination experienced by those experiencing homelessness as reported in the focus group can have impacts on their overall health and well-being making it difficult to utilise healthcare [30]. Tri-morbidities including co-occurring mental illness and substance misuse disorders is prevalent among this population, which can further aggravate the stigma associated with homelessness. Therefore, the health and social care workforce need to be trained, skilled and equipped to provide high quality health care with a focus on trauma-informed care.

Peer advocacy (experts by experience) encourages people experiencing homelessness to engage with and advocate for homeless populations. Peer advocates have the skills and experience to communicate with people experiencing homelessness as evidenced by findings from the focus group. They reported that they could empathise with the those exepriencing homelessness and supported them with navigating the health and social care systems. Peer advocacy been shown to increase engagement with with health services whilst also creating an opportunitiy to offer a wider perspective on other issues (benefits and housing) [31, 32]. Findings from Groundswell and the Young Foundation (2016) suggest that Homeless Health Peer Advocacy results in better health outcomes at a reduced cost to the NHS [33]. Attendance at outpatient appointments increased by 130% during the intervention, leading to more engagement, referrals and adherence to courses of treatment. NHS secondary care costs decreased by 42% after the intervention [33].

### Strengths and limitations

This is one of few studies utlitising mixed methods to gain an undertanding of oral health needs of those experiencing homelessness in London. The voice of those who have experienced homeless was reflected in findings from focus group with peer advocates. The mapping exercise and oral health needs assessment was conducted in the London region including a small sample of those experiencing homelessness during the pandemic. This study only included adults and did not include homeless families. It would have been helpful to have a larger sample size from multiple regions within the UK, however London has the highest rate of homelessness in England. Other studies which have been conducted have also relied on smaller sample sizes in specific regions in the UK [16, 17].

There was no clinical component to this oral health needs assessment, and therefore normative need could not be assessed when compared to other studies [16]. However, clinical examination was deemed to be inappropriate given the clinical vulnerabilies of this population to COVID-19 and reduced provison of routine dental care due to pandemic restrictions in England.

Language barriers are also known to be a common challenge in homeless populations and there was no access to translators and intrepretors when conducting the questionnaire survey due to COVID restrictions. The profile of homelessness has changed over time and some of those who are ‘entrenched rough sleepers’ may have not been represented in this sample.

Definitions of homelessness can be challenging with no agreed definitions globally [34]. This is exacerbated by limited surveillance data on health, housing, and social care needs, which isn’t routinely collected as it is for the general population. However, for our study, we defined homelessness as those who were sleeping rough on the streets representing the extreme form of homelessness. The sample did not include wider definitions of homelessness such as those residing in temporary accommodation, sofa surfers, and those at risk of homelessness. Defining and counting who is homeless is challenging and understanding and monitoring the needs of this heterogenous population to inform national policies can therefore be difficult to disentangle [35]. Ideally, a more comprehensive data set which includes immigration status, education and health status would have been helpful, however, this could not be achieved.

## Conclusion

Homelessness is a global public health challenge with significant impacts on individuals and their quality of life with its associated social and economic costs. This study has demonstrated the significant oral health needs of those experiencing homelessness across London with impacts on psycho-social, functional and psychological well-being. Dental services alone will not tackle oral health inequalities. Therefore, providing housing solutions with wrap around health and social care could address the structural determinants and thereby improve oral health outcomes for this vulnerable population. Housing and health are fundamentally intertwined, both being a human right. Considering the impacts of oral health on health related-quality of life and the vulnerability of this population, the importance of oral health should not be under-estimated. Evidence-based approaches are readily available to inform and deliver a homelessness prevention plan across the life course for the benefits of individuals and societies; there is no room for homelessness in the 21st Century.

### Supplementary Information

**Additional file 1.**

## Data Availability

The datasets used and/or analysed during the current study are available from the corresponding author on reasonable request.
