# Supplementary material for: Oral health of people experiencing homelessness in London: a mixed methods study
Source: BMC Public Health. 2023 Sep 4;23:1701. doi: 10.1186/s12889-023-16648-x (PMC10476388; doi:10.1186/s12889-023-16648-x)
Supplement: Supplementary file 1 — Additional file 1. [file 12889_2023_16648_MOESM1_ESM.pdf]

Public Health England

Protecting and improving the nation's health

# A survey to understand the dental needs of homeless populations in London

**Why we are doing the survey?**

We would like to have your views and experiences of your own dental health and how you feel about visiting an NHS dentist. This information will be valuable in trying to improve dental services for you.

**Confidentiality**

We do not ask for your name in this survey. Any information used in future publication and reports will not contain any confidential details

**How long it would take to complete**

This survey should take around 15 minutes to complete.

Thank you for your time

**About you**

**1. What is your gender?**

- Male ☐
- Female ☐
- Other ☐
- Prefer not to say ☐

**2. What is your age?**

- Under 25 ☐
- 25 – 34 years old ☐
- 35 – 44 years old ☐
- 45 – 54 years old ☐
- 55 – 64 years old ☐
- 65 years or older ☐

**3. What is your ethnicity?**

**White:**

- British ☐
- Irish ☐
- Gypsy or Irish Traveller ☐
- Any other please specify……………………………………………………… ☐

**Mixed:**

- White and Black Caribbean ☐
- White and Black African ☐
- White and Asian ☐
- Any other please specify……………………………………………………. ☐

**Asian / Asian British:**

- Indian ☐
- Pakistani ☐
- Bangladeshi ☐
- Chinese ☐
- Any other please specify……………………………………………………… ☐

**Black / African / Caribbean / Black British:**

- African ☐
- Caribbean ☐
- Any other please specify……………………………………………………… ☐

**Other ethnic group:**

- Arab ☐
- Any other please specify……………………………………………………… ☐

## Health Behaviours

4. **Do you usually have sugar when drinking hot drinks?**
   - Yes ☐
   - No ☐
   - Do not drink hot drinks ☐

5. **On average, how often do you eat chocolates or sweets?**
   - 6 or more times a week ☐
   - 3-5 times a week ☐
   - 1-2 times a week ☐
   - Less than once a week ☐
   - Rarely or never ☐

6. **On average, how often do you have fizzy drinks, fruit juice or soft drinks like squash (not including diet or sugar-free drinks)?**
   - 6 or more times a week ☐
   - 3-5 times a week ☐
   - 1-2 times a week ☐
   - Less than once a week ☐
   - Rarely or never ☐

7. **How often do you brush your teeth?**
   - Less than once a day ☐
   - Once a day ☐
   - Twice a day ☐

8. **Do you use methadone?**
   - Yes ☐
   - No ☐

9. **If Yes, is the methadone you use sugar-free?**
   - Yes ☐
   - No ☐
   - Unsure ☐

10. **Do you smoke?**
   - Yes ☐
   - No, past smoker ☐
   - Never smoked ☐

**About your dental health**

**11. How concerned are you about your dental health at this time?**

- Very concerned ☐
- Fairly concerned ☐
- Not very concerned ☐
- Not at all concerned ☐

**12. Do you currently have any of the following?**

- Tooth abscess ☐
- Bleeding or swollen gums ☐
- Loose teeth ☐
- Tooth ache or pain in your mouth ☐
- Sensitive teeth ☐
- Other

If you ticked ‘other’ please give details

| **In the last 12 months have you:** | Never | Hardly ever | Occasionally | Fairly Often | Very Often |
|---|---|---|---|---|---|
| 13. had trouble **pronouncing any words** because of problems with your teeth, mouth or dentures? | ☐ | ☐ | ☐ | ☐ | ☐ |
| 14. felt that your **sense of taste** has worsened because of problems with your teeth, mouth or dentures? | ☐ | ☐ | ☐ | ☐ | ☐ |
| 15. had **painful aching** in your mouth? | ☐ | ☐ | ☐ | ☐ | ☐ |
| 16. found it **uncomfortable to eat any foods** because of problems with your teeth, mouth or dentures? | ☐ | ☐ | ☐ | ☐ | ☐ |
| 17. been **self-conscious** because of your teeth, mouth or dentures? | ☐ | ☐ | ☐ | ☐ | ☐ |
| 18. **felt tense** because of problems with your teeth, mouth or dentures? | ☐ | ☐ | ☐ | ☐ | ☐ |
| 19. had a **diet** that is **unsatisfactory** because of problems with your teeth, mouth or dentures? | ☐ | ☐ | ☐ | ☐ | ☐ |
| 20. been a bit **embarrassed** because of problems with your teeth, mouth or dentures? | ☐ | ☐ | ☐ | ☐ | ☐ |
| 21. been **totally unable to function** because of problems with your teeth, mouth or dentures? | ☐ | ☐ | ☐ | ☐ | ☐ |

**Visiting the dentist**

**22. When did you last go to the dentist?**

- In the last 6 months ☐
- 6 months to a year ago ☐
- 1 to 2 years ago ☐
- 2 to 5 years ago ☐
- 5 to 10 years ago ☐
- Over 10 years ago ☐
- I don't remember ☐

**23. What was the reason you last visited a dentist for?**

- Routine check-up ☐
- Emergency or urgent treatment ☐
- Other reason ☐

If you ticked 'other' please give details

**24. Where was your last dental appointment?**

- High street dental practice ☐
- Community health centre ☐
- Hospital ☐
- Mobile dental van ☐
- Crisis UK ☐
- Other ☐

If you ticked 'other' please give details

**25. Did the dentist treat you with dignity and respect?**

- Yes ☐
- No ☐

**26. If you have not been to the dentist in the last 2 years, why not?**

- Nothing wrong with my teeth ☐
- Can't find a dentist ☐
- I can't afford NHS charges ☐
- I haven't got time to go ☐
- I am afraid of going to the dentist ☐
- Keep forgetting / haven't got around to it ☐
- I'm too embarrassed to go to the dentist ☐
- I've had a bad experience with a dentist ☐
- Other reason ☐

If you ticked 'other reason' please give details

**27. Where do you prefer to have your dental treatment done?**

- At a high street dentist ☐
- At a mobile van ☐
- Community dental service ☐
- Hospital dental service ☐
- In a place with other services e.g. GP ☐
- I don't mind ☐
- Other ☐

**If 'other' please give more details below**

**28. When would you prefer your dental appointment to be**

- Morning ☐
- Lunchtime ☐
- Afternoon ☐
- Evening ☐
- I don't mind ☐

**29. How would you prefer to book your dental appointment?**

- Telephone ☐
- By text ☐
- In person ☐
- Via a keyworker/case worker ☐

**30. How would you prefer dental appointment reminders be sent?**

- Telephone ☐
- By text ☐
- In person ☐
- Via a keyworker/case worker ☐

**31. When would you prefer dental appointment reminders be sent?**

- The day before the appointment ☐
- The day of the appointment ☐
- The hour before the appointment ☐
- Other ☐

If you ticked 'other' please give details

**COVID-19 (Coronavirus) and your dental health**

**32. Have you had noticed pain in your teeth or mouth since 'lockdown'**

Yes ☐
No ☐

**If 'Yes' did you get an appointment with a dentist?**

Yes ☐
No ☐

**If 'Yes' how did you make this appointment?**

With your NHS dentist ☐
NHS 111 ☐
Walk in service ☐

**If 'No' why did you not get an appointment?**

I did not know how to get one ☐
I was given advice or medicine to help ☐
I did not want to wait ☐
Appointment offered was not suitable ☐

**33. Do you have any suggestions to help us improve dental services?**

**Thank you for taking the time to complete this survey. If you wish to get some dental advice or to see a dentist, please call 020 33168353**

**A member of the community dental team will help to arrange an appointment for you with the community dental service.**
